# Delphinidin-3-sambubioside from Hibiscus sabdariffa. L attenuates hyperlipidemia in high fat diet-induced obese rats and oleic acid-induced steatosis in HepG2 cells

**DOI:** 10.1080/21655979.2021.1950259

**Published:** 2021-07-20

**Authors:** Qionghua Long, Hongyan Chen, Wenhui Yang, Li Yang, Lijuan Zhang

**Affiliations:** a Department of General Medicine, Yanan Hospital of Kunming, Kunming, China; b Department of Neurology, Yanan Hospital of Kunming, Kunming, China

**Keywords:** Hibiscus sabdariffa, lipid metabolism, hyperlipidemia, diet-induced obesity

## Abstract

*Hibiscus sabdariffa*. L is folk medicine that is often used for its hypolipidemic and antihypertensive effects; however, the active compound responsible for its anti-obesity effect is presently unknown. Delphinidin-3-sambubioside (Dp3-Sam) is an anthocyanin, was extracted from *Hibiscus sabdariffa* L. The present research aimed to investigate the role of Dp3-Sam in the prevention of hyperlipidemia in vivo and in vitro. Rats were fed with a standard chow diet (Control group) or high-fat diet (HFD and DP group) for eight weeks. Besides, HepG2 cells were stimulated with 0.2 mM oleic acid, with or without Dp3-Sam (100–200 µg/ml). Lipid profiles were measured by commercial kits. Oil Red O staining was performed to measure the hepatic and intracellular lipid levels. The key genes of lipid metabolism were measured by RT-PCR. In HFD-fed rats, Dp3-Sam reduced the body weight gain, visceral fat, and abdominal fat and decreased hepatic lipid deposits. Dp3-Sam decreased intracellular TG levels and lipid accumulation in oleic acid-treated HepG2 cells. Besides, Dp3-Sam downregulated the mRNA expression of HMG-CoA reductase (*HMGCR*), sterol regulatory element-binding protein-1 c (*SREBP-1 C*), fatty acid synthase (*FASN*), and acetyl-CoA carboxylase (*ACC*) and upregulated the mRNA expression of cholesterol 7α-hydroxylase (*CYP7A1*), carnitine palmitoyltransferase1 (*CPT1*), acyl-coenzyme A oxidase (*ACOX*), and peroxisome proliferator-activated receptor alpha (*PPARα*). Dp3-Sam up-regulated the expression of phosphorylation of AMP-activated protein kinase (pAMPK) in HFD-fed rats. Our findings indicated that Dp3-Sam possesses the potential to improve lipid metabolism dysfunction and our results offered evidence for the use of Dp3-Sam as therapy for the prevention of obesity and dyslipidemia.

## Graphical abstract

**Figure:**
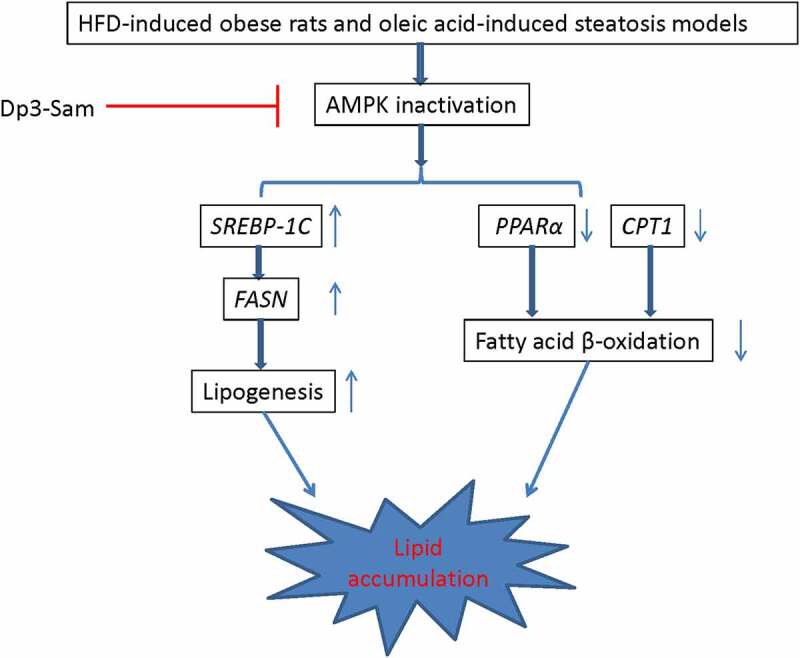

## Introduction

Growing evidence showed that obesity is a primary risk factor for non-communicable diseases, including cardiovascular, hypertension, osteoarthritis, type 2 diabetes, and cancer diseases [1,2]. And previously study has indicated that the obesity rate is significantly increased from 1975 to 2014 in the world, which has an important impact on the economy and world health [3]. Sedentary lifestyles or high-calorific diets are the main causes of obesity, which could ultimately result in hepatic lipid accumulation, such as lipid droplets, total cholesterol (TC), and triglycerides (TG) in hepatocytes [4]. The liver is a primary place for lipid metabolism; therefore, harmonious regulation in lipid accumulation and utilization is important in the treatment of metabolic disorders and hyperlipidemia [5].

The current therapies for the treatment of hyperlipidemia and obesity, such as orlistat, lorcaserin, and liraglutide, are limited by their considerable side effects [6]. Therefore, an effective and safe pharmacological therapy is indispensable. Lots of researches have shown that natural medicine plant was potential material for preventing and treating multiple diseases, including cardiovascular disease, cancer, and metabolic disease since herbal therapy had a lower incidence of side effects than chemical drug therapy [7,8].

*Hibiscus sabdariffa* L. (Hs, roselle) is a perennial herb, has been traditionally used as an herbal medicine in China [9]. It belongs to the family of Malvaceae and is widely cultivated in many countries for its medicinal and industrial applications [10]. Pre-clinical and clinical researches have shown that *H. sabdariffa* L. extract possessed anti-hypertensive, anti-diabetic, hepatoprotective, anti-oxidant, anti-obesity, and antibacterial effects [9]. Organic acids, anthocyanins, phenolic acid, and flavonoids are the main bioactive constituents in the *H. sabdariffa* [9]. The previous report has indicated that *H. sabdariffa* ethanolic, methanolic, and aqueous extracts rich in flavonoids and polyphenols exerted anti-inflammatory, antihypertension, and antihyperlipidemic activities [10]. Another research reported that *H. sabdariffa* calyces extract is rich in anthocyanins, such as cyanidin-3-sambubioside, delphinidine-3-glucoside, and delphinidine-3-sambubioside (Dp3-Sam), contributing to their antioxidant effects [11]. However, whether the Dp3-Sam exerts hypolipidemic effects and underlying mechanisms is still unclear.

We hypothesized that Dp3-Sam could prevent the progression of hyperlipidemia. Therefore, based on these researches, we used rat hyperlipidemia model induced by HFD and oleic acid-induced steatosis of HepG2 cells to explore the therapeutic potential of Dp3-Sam in the prevention of hyperlipidemia. Besides, the key genes related to lipids metabolism were measured to explore the underlying mechanism of action of Dp3-Sam.

## Materials and methods

### Determination of Dp3-Sam by high-performance liquid chromatography (HPLC)

**Figure 1.**
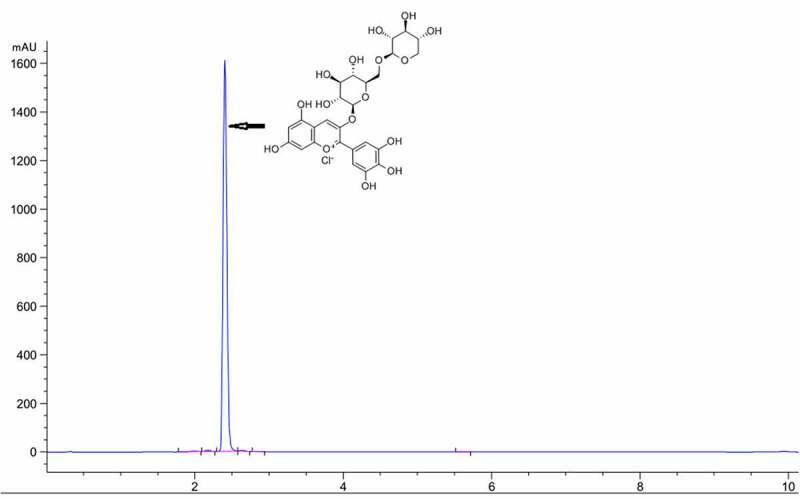
HPLC chromatogram of delphinidin-3-sambubioside (Dp3-Sam)

Dp3-Sam was obtained from Sichuan Weiqi Biological Technology Co., Ltd (Sichuan, China) and its purity was measured by a lipid chromatography (Agilent 1260), with a UV/VIS detector. An Agilent ZORBAX Eclipse Plus C18 column (4.6 × 50 mm, 3.5 µm) was used and the wavelength was 520 nm. The mobile phase consisted of solvents A (acetonitrile) and B (0.1% formic acid in water) and the mobile phase in gradient mode as follows: 0 min, 10% A; 6 min, 80%A; 10 min 80%A. The flow rate of the mobile phase was 0.6 ml/min and the injection volume was 10 µl. The HPLC purity of Dp3-Sam was 98.91% (Figure 1).

### Animals

The six-week-old of Sprague-Dawley rats were purchased from the Medical Laboratory Animal Center of Yunnan (Kunming, China). All animals were obtained free tap water and food under a controlled humidity (40–60%) and room temperature (20–22°C) with 12/12 h light-dark schedules. The animal experimental protocol was approved by the Animal Ethics Committee of Yanan hospital of Kunming.

### Animal study design and Dp3-Sam administration

A rat model of hyperlipidemia was induced according to the previous report with minor modification [12]. After seven days of acclimatization. All rats were classified into four groups (n = 8) as follows: Control group (Control): rats were fed normal chow diet for eight weeks; high-fat diet group (HFD): rats were fed HFD for eight weeks; a low dose of Dp3-Sam group (LDP): rats were fed with HFD and gavaged with Dp3-Sam (15 mg/kg body weight) daily for eight weeks; high dose of Dp3-Sam group (HDP): rats were fed with HFD and gavaged with Dp3-Sam (30 mg/kg body weight) daily for eight weeks. The formula of the experimental diets was present in Table 1. During the experiment, food intake and body weight were measured. The food efficiency ratio was obtained using the following formula: weight gain/food intake × 100%. After 12 h of fasting, rats were anesthetized using ketamine and xylazine. The blood was obtained from the inferior vena cava. The visceral fat, abdominal fat, and liver tissue were harvested, and stored at −80°C for further assay. The liver index was measured using the following formula: liver weight/body weight × 100%.

**Table 1.** The formula of the experiment diets (%)

Ingredient	High-fat diet	Standard chow
Total protein (%)	-	28.5
Crude fat (%)	-	13.5
Carbohydrates (%)	-	58
Standard chow (%)	48%	-
Soybean oil (%)	8%	-
Sweetened condensed milk (%)	44%	-

### Measurement of fecal lipids

At the end of the experiment, rat feces were collected, lyophilized, and weighted. Then, the feces were crushed and about 0.4 g of powder was ultrasonically extracted with 20 mL of chloroform/methanol (2:1, v/v) for 0.5 h. Finally, we collected the supernatant and fecal lipids were obtained using the vacuum drying method [13].

### Biochemical analysis

The serum sample was prepared by centrifugation of the blood sample at 4000 rpm for 10 min. The liver tissue was homogenized in saline solution and centrifuged at 4000 rpm for 10 min. We collected the supernatant for biochemical analysis. The levels of serum TC, TG, low-density lipoprotein cholesterol (LDL-C), high-density lipoprotein cholesterol (HDL-C), hepatic TC, and hepatic TG were assayed by commercial kits based on the manufacturer’s protocol (Jiancheng Bioengineering Institute, Nanjing, China).

### Histopathological analysis

The lipid deposition of hepatic tissues was observed by Oil Red O staining. Briefly, freshly collected hepatic tissues were fixed in 4% paraformaldehyde for 72 h. After dehydration with ethanol, the hepatic tissues were embedded in paraffin. Then, the embedded hepatic tissues were cut into 6 µm-thick sections and incubated with 10% formalin at room temperature for 0.5 h, then stained with Oil Red O reagent for 20 min. The stained section was observed using a microscope (Olympus, Tokyo, Japan) and enlarged 200× to evaluate lipid deposition.

### Cell culture and Dp3-Sam treatment

A HepG2 cells model of hyperlipidemia was induced according to the previous report with minor modification [14]. Human hepatocyte (HepG2) cells were purchased from the China Cell Line Bank (Beijing, China) and were cultured in Dulbecco’s modified eagle medium (1% penicillin-streptomycin and 10% fetal bovine serum) with 5% CO_2_ in a humidified atmosphere at 37°C. After reaching about 80% confluence, HepG2 cells were starved and then stimulated with oleic acid (0.2 mM), with or without Dp3-Sam (0–500 µg/ml), for 24 h.

### Measurement of cell viability

Cell viability was measured using the cell counting kit-8 colorimetric assay. Briefly, the HepG2 cells were seeded in 96-well plates (5 × 10^3^ cells/well) and cultured for 24 h. Subsequently, cells were stimulated with oleic acid (0.2 mM), with or without Dp3-Sam (0–500 µg/ml), for 24 h. Cell Counting Kit-8 (Dojindo Molecular Technologies, Inc., Japan) was used to measure the cell viability according to the manufacturer’s protocols. Besides, HepG2 cells were collected for TG measurement by the commercial kit (Nanjing Jiancheng Bioengineering Institute, Nanjing, China) following the manufacturer’s protocols.

### Oil Red O staining and assay of lipid accumulation

HepG2 cells were stimulated with oleic acid (0.2 mM), with or without Dp3-Sam (100 and 200 µg/ml), for 24 h. After that, HepG2 cells were fixed in formalin solution (10%) for 0.5 h and stained by Oil Red O solution for 0.5 h. After the stained cells were washed with PBS and cells were observed by an inverted microscope. 60% isopropanol was used to extract the lipid droplets and measured the lipid accumulation colorimetrically at 510 nm.

### Real-time quantitative polymerase chain reaction (RT-PCR)

Total RNA was isolated from hepatic tissue using TRIzol reagent based on the suppliers’ specifications (Invitrogen, CA, USA). The cDNA synthesis was conducted using the GoScript Reverse Transcription kit based on the suppliers’ specifications (Thermo, USA). The mRNA levels were estimated by RT-PCR using an SYBR Green qPCR Master Mix kit based on the suppliers’ specifications (Takara, Dalian, China). The qPCR was performed by an RT-PCR detection system (Bio-Rad, Hercules, CA). The PCR reaction conditions were performed as follows: 95°C for 2 min; 40 cycles of 95°C for 20 s, 60°C for 30 s and 70°C for 15 s. The primer sequences used to amplify mRNA were shown in Table 2. Fold expression relative to the reference gene (GAPDH).

**Table 2.** Primer sequences for quantitative real-time RNA

Genes	Forward primer	Reverse primer
Rat *ACC*	5'-CTGCTGGAGACCGAAAGCTT-3’	5'-CAACATGGTGTCAGGACGTTCT-3’
Rat *FASN*	5'-AGCCCCTCAAGTGCACAGTG-3’	5'-TGCCAATGTGTTTTCCCTGA-3’
Rat *SREBP-1 C*	5'-CCCTGCGAAGTGCTCACAA-3’	5'-GCGTTTCTACCACTTCAGGTTTCA-3’
Rat *HMGCR*	5'-TGTTCACCGGCAACAACAAGA-3’	5'-CCGCGTTATCGTCAGGATGA-3’
Rat *CYP7A1*	5'-TTCTCAACGATACACTCT-3’	5'-CTCCATTCACTTCTTCAG-3’
Rat *CPT1*	5'-ACCGCCACCTCTTCTGCCT-3’	5'-AGTTCCACCTGCTGCTGAG-3’
Rat *ACOX*	5'-TTACATGCCTTTGTTGTCCCTATC-3’	5'-CGGTAATTGTCCATCTTCAGGTA-3’
Rat *PPARα*	5'-GGAAACTGCCGACCTCAAAT-3’	5'-AACGAAGGGCGGGTTATTG-3’
Rat *GAPDH*	5'-TGTGTCCGTCGTGGATCTGA-3’	5'-CCTGCTTCACCACCTTCTTGAT-3’
Human *FASN*	5'-GGGATGAACCAGACTGCGTG-3’	5'-TCTGCACTTGGTATTCTGGGT-3’
Human *SREBP-1 C*	5'-GCGCCTTGACAGGTGAAGTC-3’	5'-GCCAGGGAAGTCACTGTCTTG-3’
Human *CPT1*	5'-CTCAGTGGGAGCGGATGTTT-3’	5'-TGCTGTCTCTCATGTGCTGG-3’
Human *PPARα*	5'-AAGCTGTCACCACAGTAGCTT-3’	5'-TTCCAGAACTATCCTCGCCG-3’
Human *GAPDH*	5'-GAGCATCCCCCAAAGTTCACAA-3’	5'-AGTGGGGTGGCTTTTAGGAT-3’

### Western blot

Liver tissues were lysed in cold RIPA buffer to extract the total proteins. Then, the bicinchoninic acid protein assay kit (Nanjing Jiancheng Bioengineering Institute, Nanjing, China) was used to measure the protein concentration. The protein (35 µg) was separated on a sodium dodecyl sulfate-polyacrylamide gel electrophoresis (SDS-PAGE) and transferred to nitrocellulose membranes (Millipore, USA). After the membranes were blocked with bovine serum albumin (5%) for 2 h at room temperature and then were incubated at 4°C overnight with the primary antibodies against phosphor-AMPK, AMPK, and β-actin, followed by the horseradish peroxidase-conjugated secondary antibody. These antibodies were obtained from Cell Signaling Technology. An enhanced chemiluminescence detection reagents (Bio-Rad, CA, USA) was used to measure the protein bands.

### Statistical analysis

Data were presented as means ± standard error of the mean (SEM). Statistical comparisons were analyzed by one-way analysis of variance (ANOVA) and data were statistically evaluated using GraphPad Prism software (GraphPad Software, Inc., La Jolla, USA). Differences were deemed significant when *P* < 0.05.

## Results

The present research aimed to investigate the beneficial effects and underlying mechanism actions of Dp3-Sam on hyperlipidemia. In our study, we hypothesized that lipid metabolism involved in Dp3-Sam’s protective effects against hyperlipidemia. And both rat and HepG2 cells model of hyperlipidemia were used to validate our hypothesis.

### The effects of Dp3-Sam on food intake, body weight, tissue weight, and food efficiency ratio

**Figure 2.**
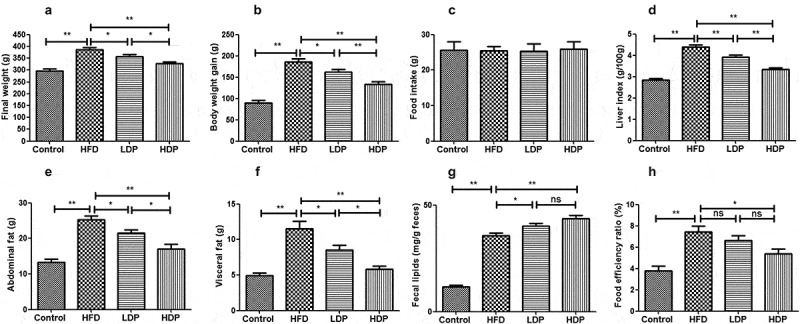
Beneficial effects of delphinidin-3-sambubioside (Dp3-Sam) on high fat diet (HFD)-induced obese rats. (a) final weight; (b) body weight gain; (c) food intake; (d) liver index; (e) abdominal fat; (f) visceral fat; (g) fecal lipids, and (h) food efficiency ratio. Data are reported as mean ± SEM. * *P* < 0.05, ** *P* < 0.01, no significant difference (ns)

A rat model of hyperlipidemia was established to investigate the effects of Dp3-Sam on hyperlipidemia in vivo. The final body weight, body weight gain, liver index, abdominal fat, visceral fat, and food efficiency ratio of the HFD group were higher when compared with those of the control group (Figure 2). Administration of LDP or HDP inhibited the increase of liver index, body weight gain, final body weight, abdominal fat, visceral fat, and food efficiency ratio induced by HFD. Additionally, HDP showed the better effect than LDP (*P* < 0.01 or *P* < 0.05). As shown in Figure 2c, no obvious differences were observed among all groups for food intake, showing that the loss in body weight was attributed to the reduction of fat accumulation and not result from lower energy intake. Besides, the fecal lipid content of rats in the HFD group was higher compared to the control group (Figure 2g). After administration of LDP or HDP, the fecal lipid content was increased compared with the HFD group (*P* < 0.01 or *P* < 0.05). There was no significant difference in fecal lipid and food efficiency ratio between the LDP and HDP groups (*P* > 0.05). Our findings showed that long-term treatment of Dp3-Sam decreased lipid accumulation in HFD rats.

### Histopathological analysis

**Figure 3.**
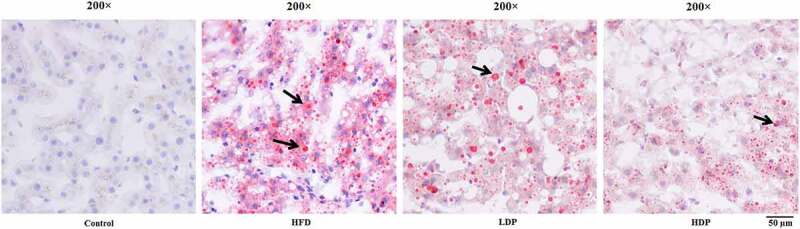
Treatment with delphinidin-3-sambubioside (Dp3-Sam) alleviates lipid deposition in HFD rats. Representative images of hepatic tissue Oil Red O staining of each group, black arrows indicated lipid droplet. Scale bar, 50 µm

Oil Red O staining was employed on hepatic sections to assess hepatic steatosis (Figure 3). The lipid droplet was not observed in the hepatic tissue section of the control group, while many lipid droplets were found in the HFD group. However, after treatment with Dp3-Sam for eight weeks, the number and degree of red lipid droplets in the LDP and HDP group were lower than those of the HFD group. The improvement was particularly significant in the HDP group, indicating that Dp3-Sam could alleviate hepatic lipid accumulation in the HFD group.

### The effects of Dp3-Sam on serum and hepatic lipid profile in HFD rats

**Figure 4.**
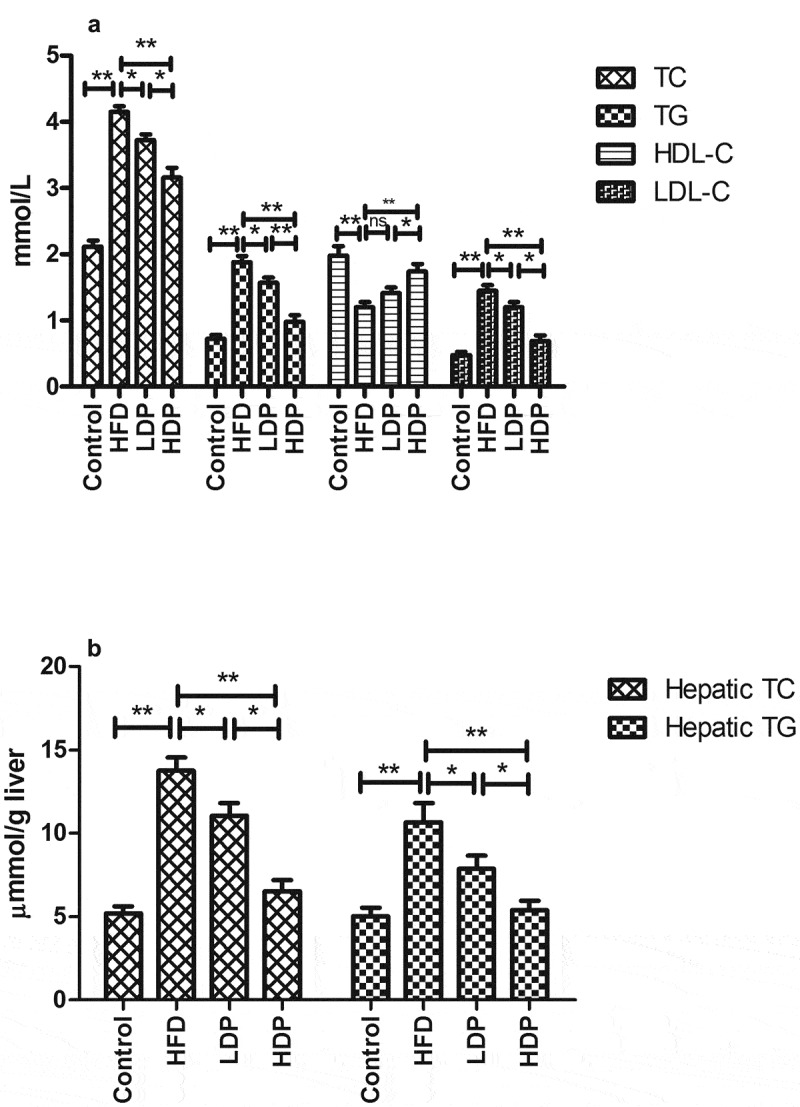
Effect of delphinidin-3-sambubioside (Dp3-Sam) on serum lipid profile (a), and hepatic lipid profile (b) in the high-fat diet (HFD)-induced obese rats. Low-density lipoprotein cholesterol (LDL-C), high-density lipoprotein cholesterol (HDL-C), triacylglycerol (TG), and total cholesterol (TC). Data are reported as mean ± SEM. * *P* < 0.05, ** *P* < 0.01, no significant difference (ns)

ELISA kits were used to measure the lipid profiles in serum and liver tissue. After HFD for eight weeks, the serum HDL-C level was decreased, while the serum LDL-C, TG, and TC levels were increased compared to those in the control group (Figure 4a). Administration of LDP or HDP for eight weeks improved these lipid profiles in the HDP group. HDP exhibited the better effect than LDP (*P* < 0.01 or *P* < 0.05). Besides, as shown in Figure 4b, after HFD for eight weeks, the hepatic TG and TC levels were increased compared with those in the control group (*P* < 0.01). However, administration of LDP or HDP for eight weeks improved the hepatic lipid profiles in the LDP and HDP groups. And HDP exhibited a better effect than LDP (*P* < 0.05).

### Effect of Dp3-Sam on mRNA expression in liver

**Figure 5.**
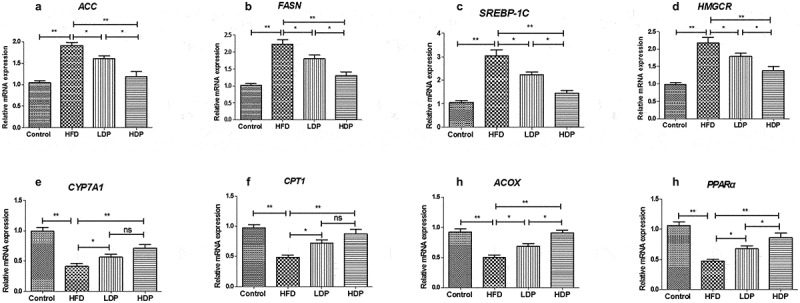
Effect of delphinidin-3-sambubioside (Dp3-Sam) on liver expression of *ACC* (a), *FAS* (b), *SREBP-1 C* (c), *HMGCR* (d), *CYP7A1* (e), *CPT1* (f), *ACOX* (g), and *PPARα* (h) in high fat diet (HFD)-induced obese rats. Data are reported as mean ± SEM. * *P* < 0.05, ** *P* < 0.01, no significant difference (ns)

As shown in Figure 5, the mRNA expressions of lipid metabolism-related genes were measured to explore the underlying mechanism actions of Dp3-Sam against lipid metabolism disorder. The mRNA expression levels of *HMGCR, FAS, SREBP-1 C*, and *ACC* were upregulated in the HFD group, while *CYP7A1, CPT1, ACOX*, and *PPARα* mRNA expression levels were downregulated in the HFD group compared to those in the control group (*P* < 0.01). However, administration of LDP or HDP downregulated the hepatic *SREBP-1 C, ACC, FAS*, and *HMGCR* mRNA expression, and upregulated the hepatic *CYP7A1, CPT1, ACOX*, and *PPARα* mRNA expression (*P* < 0.01 or *P* < 0.05) in the HFD group. Additionally, HDP showed a better effect than LDP (*P* < 0.05). There was no significant difference in *CYP7A1* and *CPT1* between the LDP and HDP groups (*P* > 0.05).

### Effect of Dp3-Sam on activation of AMPK in HFD rats

**Figure 6.**
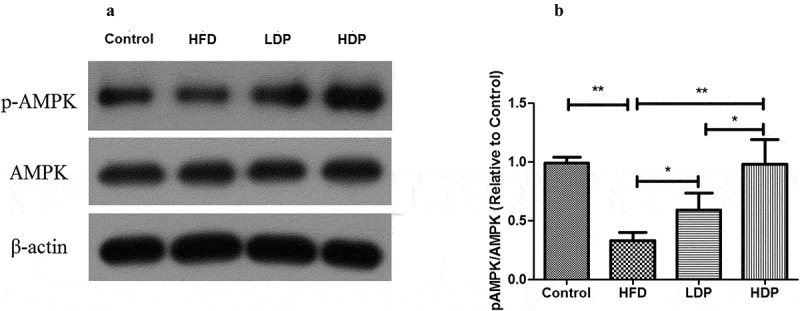
Effect of delphinidin-3-sambubioside (Dp3-Sam) on the expression of AMPK in high fat diet (HFD)-induced obese rats. The expression of AMPK and pAMPK in hepatic tissues were measured by western blot. Data are reported as mean ± SEM. * *P* < 0.05, ** *P* < 0.01, no significant difference (ns)

We measured the protein expression of pAMPK/AMPK to investigate the role of Dp3-Sam on the hepatic AMPK pathway. As shown in Figure 6, the phosphorylated AMPK to AMPK ratio was declined in the HFD group compared with the control group (*P* < 0.01). Administration of HDP increased the ratio back to the normal level. These findings showed that Dp3-Sam regulated lipogenesis-related genes via the activation of the AMPK pathway.

### Cell viability of Dp3-Sam

**Figure 7.**
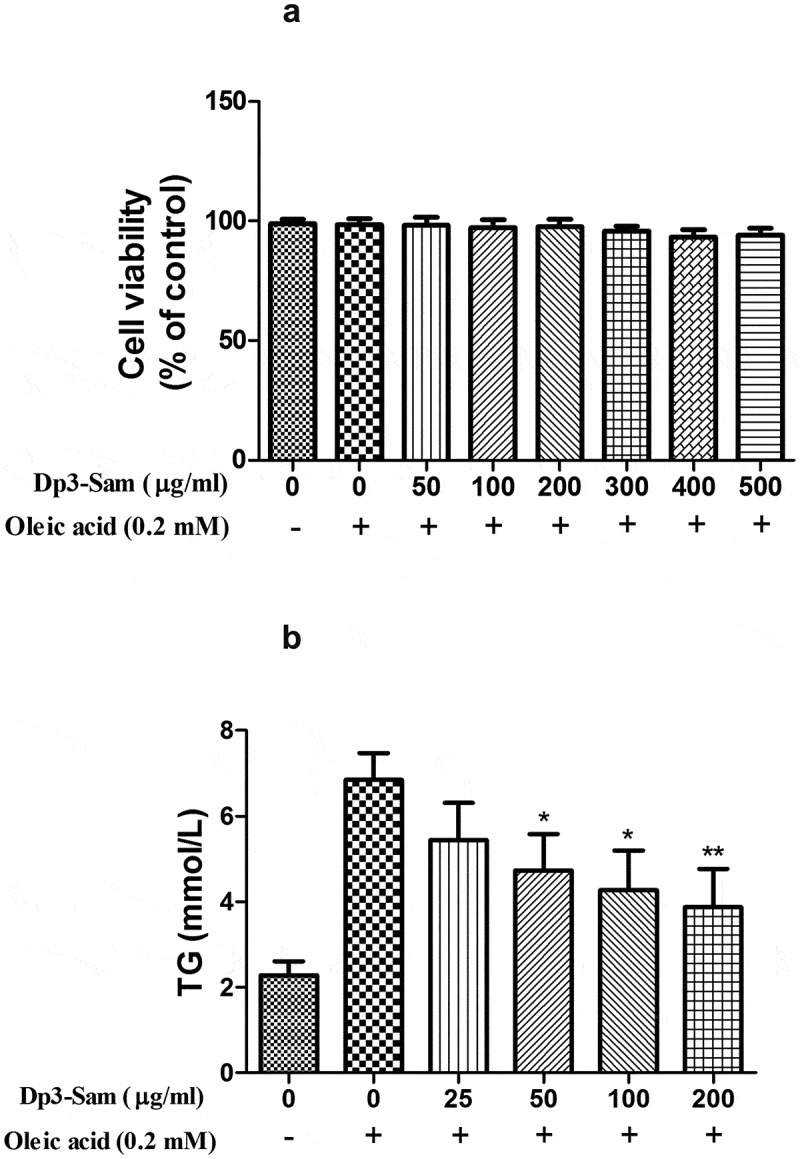
Effect of delphinidin-3-sambubioside (Dp3-Sam) on cell viability and TG levels in oleic acid-treated HepG2 cells. (a) Cell viability was measured by MTT. (b) The levels of TG in HepG2 cells were assayed. HepG2 cells were stimulated with oleic acid (0 or 0.2 mM) and treated with different concentrations of DP (0–500 µg/ml) for 24 h. Data are reported as the mean ± SEM. * *P* < 0.05, ** *P* < 0.01 vs. oleic acid group

A HepG2 cells model of hyperlipidemia was established to further validate the protective actions of Dp3-Sam on hyperlipidemia in vitro. The effects of oleic acid (0.2 mM) plus different concentrations of Dp3-Sam (0–500 µg/ml) on cell viability were investigated (Figure 7a). The data showed that oleic acid (0.2 mM) plus Dp3-Sam (0–500 µg/ml) had no cytotoxic effect.

### Effect of Dp3-Sam on intracellular TG level in oleic acid-treated HepG2 cells

After treated with oleic acid (0.2 mM), the intracellular TG level was higher than those of the unstimulated cells (Figure 7b). Dp3-Sam treatment prevented oleic acid-induced TG accumulation in a concentration-dependent manner (25–200 µg/ml). Therefore, the effect of Dp3-Sam on lipid metabolism in vitro was evaluated using concentrations between 100 and 200 µg/ml.

### Effect of Dp3-Sam on lipid accumulation in oleic acid-treated HepG2 cells

**Figure 8.**
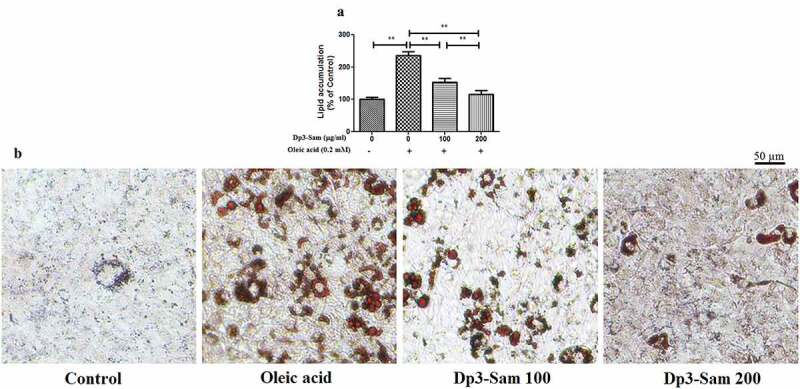
Effect of delphinidin-3-sambubioside (Dp3-Sam) on lipid accumulation in oleic acid-treated HepG2 cells. (a) Lipid accumulation was colorimetrically assayed at 510 nm. (b) Observing the Oil Red O stained cells by a microscope. Scale bar, 50 µm. HepG2 cells were stimulated with oleic acid (0 or 0.2 mM) and treated with different concentrations of DP (100 or 200 µg/ml) for 24 h. Data are reported as the mean ± SEM. * *P* < 0.05, ** *P* < 0.01 vs. oleic acid group

After treated with oleic acid (0.2 mM), the lipid accumulation was higher than those of the unstimulated cells (Figure 8a). Dp3-Sam treatment prevented oleic acid-induced lipid accumulation in a concentration-dependent manner (100–200 µg/ml). Besides, HepG2 cells exposed to the oleic acid showed a significant increase in lipid droplets compared to the unstimulated cells (Figure 8b). Dp3-Sam treatment suppressed oleic acid-induced lipid accumulation in a concentration-dependent manner (100–200 µg/ml).

### Effect of Dp3-Sam on lipid metabolism in vitro

**Figure 9.**
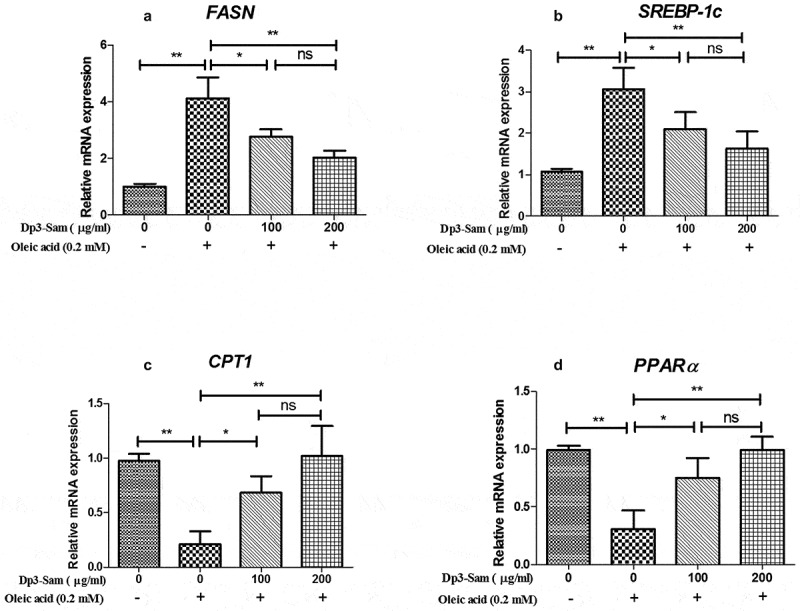
Effect of delphinidin-3-sambubioside (Dp3-Sam) on lipid metabolism in HepG2 cells. HepG2 cells were stimulated with oleic acid (0 or 0.2 mM) and treated with different concentrations of DP (100 or 200 µg/ml) for 24 h. The mRNA levels of *FASN* (a), *SREBP-1 c* (b), *CPT1* (c), and *PPARα* (d) were measured by qRT-PCR. Data are reported as mean ± SEM. * *P* < 0.05, ** *P* < 0.01, no significant difference (ns)

The genes involved in fatty acid β-oxidation and lipogenesis were measured to explore the underlying mechanism actions of Dp3-Sam against hyperlipidemia in vitro. As shown in Figure 9, our findings showed that the mRNA expression levels of *FASN* and *SREBP-1 c*, were down-regulated by Dp3-Sam treatment compared with oleic acid-treated HepG2 cells. Besides, the mRNA expression levels of *CPT1* and *PPARα* were up-regulated by Dp3-Sam treatment compared with oleic acid-treated HepG2 cells. These findings indicated that Dp3-Sam could inhibit lipid accumulation via improving lipid metabolism.

## Discussion

Long-term intake of HFD is deemed to one of the primary risk factors for obesity, cardiovascular disease, nonalcoholic fatty liver disease, and hyperlipidemia, hypertension, type 2 diabetes, and insulin tolerance, which results in chronic health problems [15]. Previous reports indicated that *H. sabdariffa* L. extract possessed anti-hypertensive, anti-diabetic, and anti-obesity effects [9]. Especially, the bioactive compounds, such as phenolic acid, flavonoids, and anthocyanins extracted from *H. sabdariffa* L., are potent in the inhibition of adipogenesis and lipid accumulation [16]. In the present study, we evaluated the improvement effects of Dp3-Sam on hyperlipidemia and lipid metabolism disorders in vivo and in vitro. Our findings for the first time demonstrated that Dp3-Sam decreased body weight gain and improved lipid profile in hyperlipidemia models.

Consistent with the previous reports [17,18], HFD administration for eight weeks caused hepatic lipid accumulation, which increases the liver index. Treatment with Dp3-Sam alleviated hyperlipidemia in the HFD-induced obese rats, as evidenced by an obvious decrease in lipid levels and hepatic lipid accumulation. Therefore, the results indicated that Dp3-Sam exerted potent efficacy to inhibit the initiation of pathogenesis associated with hyperlipidemia, and HDP showed a better effect than LDP.

The liver tissue is considered the primary organ for lipid metabolism, and hepatic lipid homeostasis is mediated by lots of enzymes, and mediators. *HMGCR*, a rate-limiting enzyme, plays a vital role in cholesterol synthesis, which controls cholesterol biosynthesis [19]. Besides, *FASN, ACC*, and *SREBP-1 C* play a vital role in the process of lipogenesis [20]. Our results showed that Dp3-Sam treatment suppressed the *ACC, FASN, SREBP-1 C*, and *HMGCR* expression in the liver tissue of HFD rats, indicating that lipid metabolism disorders could be improved by Dp3-Sam intervention. *CYP7A1*, another rate-limiting enzyme, is involved in the catabolism of cholesterol [20]. Previous reports showed that increased fatty acid β-oxidation results in reduced triglyceride levels in both animals and humans. And *CPT1, ACOX*, and *PPARα* play an important role in fatty acid β-oxidation [20]. Our results showed that Dp3-Sam treatment increased the *CYP7A1, CPT1, ACOX*, and *PPARα* expression in the liver tissue of HFD rats, indicating that Dp3-Sam intervention inhibited lipid accumulation via acceleration of lipid oxidation in HFD-induced hyperlipidemia rats.

Previous reports had indicated that the AMPK pathway regulates fatty acid oxidation and lipid synthesis in liver tissue [21–23]. It also has been reported that AMPK plays an important role in liver steatosis in HFD-induced obesity mice [24]. In the liver, AMPK is part of a mechanism that increases lipid oxidation and suppresses lipid synthesis via the inhibition of ACC activity [25,26]. Besides, hepatic activation of AMPK could protect against dyslipidemia in diet-induced insulin-resistant mice in part via inhibition of SREBP-1 c- and −2-dependent lipogenesis [22]. Our findings indicated that the phosphorylated AMPK to AMPK ratio was decreased in long term HFD group. And Dp3-Sam restored the ratio of pAMPK/AMPK to the normal level, which indicated Dp3-Sam inhibited hepatic lipogenesis via activation of the AMPK signaling pathway.

Moreover, we performed a cell experiment to confirm whether Dp3-Sam prevents lipid accumulation in oleic acid-treated HepG2 cells. Consistent with the previous reports [14,27], our findings confirmed that oleic acid caused lipid accumulation in HepG2 cells. Consistent with the results of our animal experiment, Dp3-Sam treatment prevented lipid accumulation in vitro. It has been reported that oleic acid caused lipid accumulation in HepG2 cells via the down-regulation of fatty acid β-oxidation and up-regulation of lipid synthesis [28]. Consistent with our in vivo data, Dp3-Sam treatment down-regulated the mRNA expression levels of *FASN* and *SREBP-1 c*, and up-regulated the mRNA expression levels of *CPT1* and *PPARα* in HepG2 cells.

## Conclusions

Overall, the in vivo and in vitro experiments demonstrated that Dp3-Sam alleviated hyperlipidemia via regulating the target genes related to fatty acid β-oxidation and lipogenesis via regulating AMPK activation. Together, those evidences suggested that Dp3-Sam could become a potential therapeutic drug in the prevention and treatment of obesity-related disease.

## Data Availability

The data that support the findings of this study are available from the corresponding author, upon reasonable request.

## Article highlights

1. Dp3-Sam attenuates hyperlipidemia in vivo and in vitro.
2. Dp3-Sam improves lipid metabolism via activation of AMPK signaling.
3. Dp3-Sam attenuates hyperlipidemia via regulating lipid metabolism-related genes.
